# Pilot study of a randomized trial to evaluate a Web-based intervention targeting adolescents presenting to the emergency department with acute asthma

**DOI:** 10.1186/s40814-017-0147-6

**Published:** 2017-06-21

**Authors:** Christine L. M. Joseph, Prashant Mahajan, Stephanie Stokes-Buzzelli, Dayna A. Johnson, Elizabeth Duffy, Renee Williams, Talan Zhang, Dennis R. Ownby, Shannon Considine, Mei Lu

**Affiliations:** 1 Department of Public Health Sciences, Henry Ford Health System, 1 Ford Place, 3E, Detroit, MI 48202 USA; 2 Pediatric and Communicable Diseases, University of Michigan , Ann Arbor, MI 48109 USA; 3 Department of Emergency Medicine, Henry Ford Health System, Detroit, MI 48202 USA; 4 Department of Medicine, Brigham and Women’s Hospital, Boston, MA 02115 USA; 5 Center for Health Communications Research, University of Michigan, Ann Arbor, MI 48104 USA; 6 Georgia Reserve, Department of Pediatrics, Augusta University, Augusta, GA 30912 USA; 7 Emergency Medicine Research, University of Michigan, Ann Arbor, MI 48109 USA

## Abstract

**Background:**

Low-income African-American adolescents use preventive medical services less frequently than their White counterparts, indicating a need for effective interventions targeting this group. Puff City is a Web-based, asthma management program for urban adolescents that has been evaluated in high school settings with promising results. The objective of this pilot was to assess the feasibility of initiating Puff City (treatment) in an emergency department setting, thereby informing the conduct of an individual randomized trial to evaluate its effectiveness compared to a generic, Web-based program (control) in preventing subsequent emergency department (ED) visits.

**Methods:**

Teens aged 13–19 years presenting with acute asthma to two urban EDs within the study period were eligible. Subsequent ED visits were collected using the electronic medical record. A priori indication of a potential intervention effect was *p* < 0.20.

**Results:**

Of the 121 teens randomized (65 treatment, 56 control), 86.0% were African-American and 44.6% male, with the mean age = 15.4 years. Computer ownership was reported by 76.8% of teens. Overall, 64.5% of teens completed >3 of 4 sessions and 90% completed the 12-month survey. At 12 months, the treatment group showed a trend toward fewer ED visits than controls (33.8 versus 46.4%), *p* = 0.15.

**Conclusions:**

Results indicate the feasibility of enrolling at-risk adolescents in ED settings and set the stage for a large, pragmatic trial using a technology-based intervention to reduce the burden of pediatric asthma.

**Trial registration:**

ClinicalTrials.gov, NCT01695031

## Background

According to the National Surveillance of Asthma: United States, 2001–2010 Report, death rates for asthma are higher for African-American adolescents aged 15–19 than for Whites of the same age and for younger African-American children [1]. Hospitalizations and emergency department (ED) visits are higher as well. The reasons for these disparities are multifaceted and complex. Asthma tends to concentrate in vulnerable and disadvantaged communities, where residents are often exposed to social threats, such as stress (discrimination), violence, and environmental hazards, such as chemical and air pollution [2, 3]. Youth in these communities also experience challenges in accessing high-quality preventive care, perhaps exemplified by the comparatively higher rates of asthma-related acute care visits (e.g., hospitalizations, ED visits) observed for African-American versus White adolescents aged 15–19 years of age [1]. Preventive care often offers more opportunities for asthma education and the continuity of care that encourages patient-provider communication and partnership [4].

Experts agree that interventions promoting these concepts should target high-risk populations, including persons with low socioeconomic status (SES) and those with poor access to care [5]. However, the current medical literature provides few strategies for connecting with high-risk groups of urban adolescents with inadequately controlled asthma. Acute care settings represent a promising means of identifying and intervening upon asthma patients with the greatest need.

Puff City is a Web-based, asthma management program for urban adolescents that has been evaluated in Detroit Public High Schools [6, 7]. Results of school-based randomized controlled trials were promising [6, 7]. Given the positive results of Puff City, we explored its dissemination to different settings. The objective of this pilot study was to determine the feasibility of conducting a pragmatic randomized trial to evaluate the effectiveness of an online, ED-initiated asthma management intervention designed to reduce asthma-related morbidity among urban teenagers with uncontrolled asthma. The pragmatic trial approach can be used to measure “real-world” effectiveness of Puff City and accelerate dissemination. Recently, we described the recruitment experience for this trial [6, 7]. In this paper, we characterize the randomized participants, report on participant compliance with the pilot study protocol, and describe the potential intervention effect of Puff City on selected outcomes, including ED visits and asthma control, as proof of concept that this study was feasible and the intervention could influence behavior change.

## Methods

This was a pilot study for a pragmatic, randomized, and controlled phase II trial of the Puff City asthma management program. The patient was the unit of randomization. The trial had two arms. *Arm 1* (intervention) was standard care + an online, computer-tailored asthma management intervention with behavioral assessments at each of four education sessions designed to be no less than 1 week apart and followed by a booster session delivered 6 months from baseline. *Arm 2* (control) was standard care + access to existing asthma informational websites that were non-tailored and provide generic asthma education. The control arm also had behavioral assessments at each of four sessions and at 6 months. The recruitment goal was 120 patients. This pilot study was designed to study feasibility and to determine whether an early indication of benefit from Puff City was apparent.

In 2001, Puff City (HL68971-01) was created under collaboration with the University of Michigan Center for Health Communications Research (UM-CHCR). The UM-CHCR also provided technical expertise for the Web-based intervention. Data was collected through the Henry Ford Health System (HFHS) Oracle Clinical remote data capture (RDC) system. All aspects of this project were approved by the Henry Ford Health System (HFHS; IRB# 6867), University of Michigan (IRB# HUM00066548), and Wayne State University-Detroit Medical Center (IRB# 093712B3E) institutional review boards. Two emergency department sites, HFHS and Children’s Hospital of Michigan (CHOM), participated in the trial.

### The Puff City Program

The development of Puff City has been discussed in previous publications [6, 7]. Briefly, requirements included high-speed Internet connection, a Web browser, Flash animation capabilities, and a computer with audio capabilities. The program comprised a baseline survey and four computer-tailored educational sessions. The sessions had an overall urban theme, i.e., with backdrops and included a density of urban structures, similar to landscapes of Detroit or Chicago, with persons of color (e.g., African-American, Latino) prominently featured. The sessions addressed asthma management as well as psychosocial issues, including smoking, depression, perceived emotional support, and lack of insurance/primary care physician. Professional character voices and street-wise dialog were used to capture the attention of users. A radio DJ delivered the scientifically evidence-based advice that was tailored to each teen. Each interactive session lasted 15–30 min, depending upon the number of issues reported by the teen (e.g., sharing medications, depressed, smoking, non-adherent). Puff City included a 6-month booster session. All Puff City surveys were voiced-over to accommodate literacy limitations.

The program focused on three behaviors: controller medication adherence, keeping a rescue inhaler nearby, and smoking reduction/cessation. Theory-based health messages and information on asthma control were presented in reference to these three core behaviors and allowed the delivery of information both central and peripheral to the behavior, including information on trigger avoidance, device usage (e.g., how to use a metered dose inhaler, diskus, turbuhaler, spacer, etc.), and basic asthma physiology. Details on the theories applied in Puff City have appeared in previous publications [6, 7]. Briefly, health messages and information based on theoretical models and approaches to behavior change relevant to asthma control (e.g., Health Belief Model, Attribution Theory, Motivational Interviewing) were presented for these three behaviors, allowing the delivery of information both central and peripheral to the behavior [7–10]. To apply these theories, Puff City used tailoring [8]. Tailoring is the “assessment and provision of feedback based on information that is known or hypothesized to be most relevant for each individual participant of a program” [8]. One of the main theories used in Puff City includes the Transtheoretical Model (TTM), which describes the cognitive and behavioral processes that individuals undergo in relation to changing behavior. According to the TTM, about 40% of precontemplators are “resistant” or will not exhibit behavior change without application of strategies more intense than usual. We reasoned that if potentially resistant teens could be identified early in the program, we could apply a more intense tailoring strategy through submodels and increase the probability of behavior change [9, 10].

The components of Puff City included (a) an introductory message (“Welcome to the City”), (b) assessment of current medications and how to use medication delivery devices (e.g., diskus), (c) assessment of the three core behaviors, an opportunity to select a behavior to work on, (d) comparison of perceived risk versus actual risk of a future asthma attack, (e) problem-solving, an opportunity to select values, and messages to address situational difficulties (when is it hard to use my inhaler?), and (f) session summary and good-bye. Behavior change was supported by health messages incorporating the behavioral theories mentioned above and using approaches such as motivational interviewing, as well as feedback that was normative (compared to others your age) and ipsative (compared to your last visit) delivered throughout the four sessions. Puff City also had content on when to seek medical help, overuse of beta-agonists, and scenarios to allow problem-solving. A special medication module with visual aids helped teens identify their current asthma medications. Communication with parents and providers was encouraged throughout Puff City.

### Standard of care for acute asthma in the emergency department

Adolescents with acute asthma exacerbation typically present to the ED with history of difficulty in breathing, wheezing, cough, and other symptoms such as shortness of breath and chest pain/tightness. On examination, the patients often have evidence of respiratory distress in the form of tachypnea, intercostal and suprasternal retractions, nasal flaring, and prolonged expiratory phase. Auscultation often reveals wheezing and decreased air entry in those with severe exacerbations. Functional assessments and monitoring may include arterial oxygen pressure (PaO2), arterial oxygen saturation (SaO2), peak expiratory flow (PEF) rates, and partial pressure of carbon dioxide (PCO2). A medical history will be collected on each patient, including known diagnosis of asthma, duration of symptoms, prior hospitalizations and/or intensive care admissions and intubations, and recent and historical use of asthma medications. Patients will also be asked about allergic diagnoses or symptoms (e.g., allergic rhinitis, food allergy, eczema) and other co-morbid conditions such as gastroesophageal reflux, pneumonia, or obesity. Results of these, and other assessments as needed, will help to categorize the exacerbation as mild, moderate, or severe and guide the subsequent medical treatment and subsequent decisions, including the use of corticosteroids, the decision to discharge or admit to hospital, and prevention of relapse.

### Delivering the intervention in the ED setting

Prior to this project, Puff City was evaluated in the school setting where the teen participants had access to school computers [6, 7]. To assess the feasibility of conducting a future pragmatic randomized trial to evaluate the initiation of Puff City in the ED setting, we moved the content and animations onto the Michigan Tailoring System. The Michigan Tailoring System is an open-source software package developed by CHCR to help writers develop tailored content. This software makes components of the tailoring program easier to edit and maintain. We also incorporated an email/SMS messaging system to remind participants of upcoming asthma sessions and follow-up surveys since patients would be completing sessions on a computer at home or in the community in the future trial.

### Recruitment

#### Sites

Emergency departments at two health care systems (Henry Ford Health System and Children’s Hospital of Michigan at the Detroit Medical Center) were used for this study, as were two methods of recruitment described briefly, here, and with more detail in a previous publication [11]. Children’s Hospital of Michigan (CHOM) is a for-profit, tertiary care hospital in urban Detroit, caring for most of the city’s children of indigent parents. A part of the Detroit Medical Center, CHOM is academically affiliated with Wayne State University. In 2013, a total of 403 patients aged 13–19 years made a visit to CHM Emergency Department for asthma. Henry Ford Health System (HFHS) is an 802-bed tertiary care hospital, education, and research complex located in Detroit’s New Center area. HFHS operates six hospitals in Southeastern Michigan. The HFHS Main Campus ED is one of the nine HFHS EDs in Southeastern Michigan. In 2013, a total of 110 patients aged 13–19 years visited the HFHS pediatric Emergency Department for asthma. The two health care systems are affiliated. CHOM serves as the inpatient facility for HFHS pediatric patients. Both HFHS and CHOM have participated in large, multicenter networks involving both ED-based randomized trials and observational research. Both sites have an electronic medical record (EMR) and electronic patient tracking systems in the ED, which greatly facilitate identification of potential participants. HFHS and CHM see the majority of acute asthma cases for youth in Detroit [12].

#### ED recruitment

Teens were recruited and enrolled during an ED visit for acute asthma. Recruitment tools available to research staff included remote electronic surveillance and triage systems (EMSTAT at HFHS and FIRSTNET at CHM) to identify teens potentially eligible for the study. Written informed parental permission, consent, HIPAA authorization, and assent were documented as part of the informed consent process. Caregivers could opt to refuse the caregiver portion of the study while providing permission for the teen to participate. Teens were asked to provide an email address for email reminders and a phone number for SMS reminders; however, provision of this information was optional. Owning a computer was not a requirement for participation. If the patient and caregiver were interested in participating but not willing to extend the ED visit for this purpose, research staff could arrange for the patient to return to the HFHS or CHOM at a later date and complete the enrollment process.

#### ED research and clinic staff

The research team met with ED clinicians and research staff at HFHS and CHOM to develop strategies for recruitment. CHM had research staff in the ED dedicated to patient recruitment for ongoing studies. At HFHS, research staff and several existing ED clinic staff were trained to recruit and enroll for the study. An awareness campaign for the study included fliers posted in the ED, informational letters emailed to ED physicians, Puff City as an agenda item in nursing “huddles,” and description and updates provided during divisional and practice council meetings. More detail is provided in a previous publication [11].

#### Recruitment from patient listings or “mail/phone”

After 6 months of ED recruitment, targeted recruitment benchmarks were not reached. To supplement recruitment in the ED, both sites used patient listings from administrative databases or daily logs to identify patients making recent visits to HFHS or CHOM ED with a primary or secondary ICD9 code for asthma during the recruitment period. At both sites, a letter was mailed to the patient’s home and followed by a phone call to explain the study. Interested caregivers and teens were asked to come to HFHS or CHM for an enrollment visit. At that time, research staff initiated the informed consent and assent process, followed by the enrollment process described above.

#### Inclusion/exclusion criteria

To participate, teens were 13–19 years of age and had a physician diagnosis of acute asthma at the time of the ED visit. For teens <18 years, accompaniment by a parent or legal guardian (caregiver) who provided written informed consent was required, as well as written assent from the teen. For teens >18 years of age, written consent from the teen was required. Eligibility was confirmed by ED clinical staff. Teens previously enrolled in the school-based version of Puff City were not eligible to participate in this pilot. Teens were also excluded if English was not the preferred language of the teen, as currently, Puff City was only available in English.

#### Baseline survey

After determining eligibility and obtaining consent and assent, teens completed an online baseline survey from a laptop or mobile in-house computer. The baseline survey included the Asthma Control Test™ (ACT) and the asthma self-regulatory development interview (ASRDI). The ACT is a five-question, patient-based tool designed to help physicians identify patients with poorly controlled asthma and assess the frequency of asthma symptoms, use of rescue medications, and impact of asthma on daily functioning over the course of the previous 4 weeks [13]. The ASRDI developed by Zimmerman and Bonner in 1998, theorizes that self-regulation of asthma follows a sequential path and is influenced by fundamental beliefs about the condition, perceptions of vulnerability, and the perceived ability to manage the condition [14]. The ASRDI categorizes patients into one of four phases with the lower phases representing less self-regulation [14]. The baseline survey was shortened given the limited time given for completion during an ED encounter. Items eliminated included those that could be obtained from the EMR (e.g., specialty visits) and items that could be placed in the session or follow-up surveys (e.g., depression symptoms). Sociodemographic variables obtained included age, ethnicity, race, gender, insurance status, zip code of residence (to apply census data), maternal education, and household income.

#### Randomization

Online randomization to the treatment (Puff City) or control group (generic, online asthma education) occurred when participants logged in for session 1 of the intervention/control program. For this trial, the unit of randomization was the patient. The 1:1 randomization schema was generated using the urn design [10], which was an adaptive sampling approach and provided an improvement in balance compared to blocked randomization. Prior to initiation of enrollment, we validated the randomization algorithm in collaboration with UM CHCR. Randomization was stratified by recruitment site (HFHS or WSU), gender, self-report of ED visits for asthma in the last 12 months, and ACT. As this was a study conducted among persons with acute asthma exacerbations, randomization was stratified by <15 on the ACT versus >15 (very poorly controlled), instead of <19 versus >19 [11]. The participants were blinded to the treatment assignment throughout the study.

#### Blinding

The ED and research staff were blinded to the treatment assignment, but the participant and caregiver were unblinded at the time, and after, the patient was randomized into the trial. During follow-up and retention efforts, the research staff remained blinded to the patient group assignment.

#### Online sessions and follow-up surveys

In addition to symptom frequency and health care utilization, online session surveys collected information on items required for tailoring, such as current asthma medications, smoking, controller medication adherence, and having a rescue inhaler nearby. At the initial session, survey items also included depression symptoms, perceived emotional support, and rebelliousness. Follow-up surveys collected information needed for primary and secondary endpoints, specifically items on symptoms experienced in the last 30 days, health care utilization and medication used for asthma symptoms, and items comprising the ASRDI. Teens and caregivers received incentives (cash and gift cards) for participation linked to completion of all four sessions and follow-up surveys. Targeted completion of the 6- and 12-month surveys was 70%.

#### Enrollment packet

At discharge, each patient received a Patient Enrollment Packet, containing [1] instructions on how to access Puff City sessions and follow-up surveys [2]; a letter from the ED to the teen’s high school requesting permission for the teen to use a school computer for online sessions and follow-up surveys [3]; a copy of the consent and assent forms [4]; a contact number for computer resources and/or technical problems; and [5] an incentive schedule. Participants could earn additional incentives for the final follow-up survey at 12 months.

#### Intervention delivery

The treatment group received the Patient Enrollment Packet before leaving the ED and asked to complete four online sessions (15–30 min) of Puff City within 90 days, with no less than 7 days between sessions to allow for behavior change. Treatment teens complete an online 6- and 12-month follow-up. At the 6-month survey, treatment teen responses to questions about core behaviors are compared to previous sessions. If “resistance” (no behavior change throughout the program) or “relapse” (a return to negative behaviors) was indicated, a booster session was launched and delivers additional theory-based intervention content to the teen. The booster session was about 5–10 min in length. Teens randomized to the control group will receive the Patient Enrollment Packet before leaving the ED and asked to complete four online sessions of commercial websites offering generic asthma education. After log-in, control teens are provided with a link to the control websites. Teens will be encouraged to complete the four sessions within 90 days, with a minimum of 7 days between sessions to allow for behavior change. To regulate dosage, control teens receive a “time expired” message after 30 min of browsing. This time limit corresponds to the estimated time needed to complete a tailored session. Control teens are also asked to complete the 6- and 12-month surveys, but there was no booster session at 6 months.

#### Patient safety

All enrolled patients were closely monitored for safety. Serious adverse events (SAEs) were monitored and documented during the study intervention period (about 6 months). Any SAE would prompt review by a medical expert (Dennis Ownby, MD), who was not involved in patient recruitment, for determination of possible attribution to the study intervention. The reviewer was blinded to the treatment assignment.

#### Medical record abstracting

To obtain information on health care utilization, e.g., emergency department visits, the medical charts of study participants were reviewed for the study period, defined as 1 year prior to the index visit through 12 months post index visit.

### Statistical analysis

The targeted enrollment for this pilot study was to successfully randomize 120 teens with asthma to the intervention and non-intervention cohort. This trial was designed to enroll a minimum of 54 patients per group with two groups to detect an effect size of 0.3 with 80% power, assuming a one-sided test and alpha = 0.2 [15]. The effect size of 0.3 was considered a relatively small effect as suggested by Cohen [10] for a behavioral trial. Because this was a pilot study, sample size/power calculation was predetermined based on *p* value = 0.20; however, results are focused mainly on confidence interval estimation. The primary endpoint was the number of ED visits at 12 months. Secondary endpoints included asthma control as measured by the ACT, functional status, quality of life, and behavior change. Data were evaluated for normality. The nonparametric Wilcoxon test was considered if data were not normally distributed. ACT scores were heavily skewed, and so the median score was used for illustration. The distribution for frequency of ED visits post baseline was highly skewed with a high proportion of 0 or 1. Therefore, a binary variable ED visit ≥1 was used in the analysis. Baseline variables were used to assess group balance prior to randomization. Any imbalanced variables were included in the outcome analysis. Adjusted odds ratios (OR) and corresponding 95% confidence intervals (95% CI) were calculated to measure the potential intervention effect compared to controls using multivariable logit models.

## Results

**Fig. 1:**
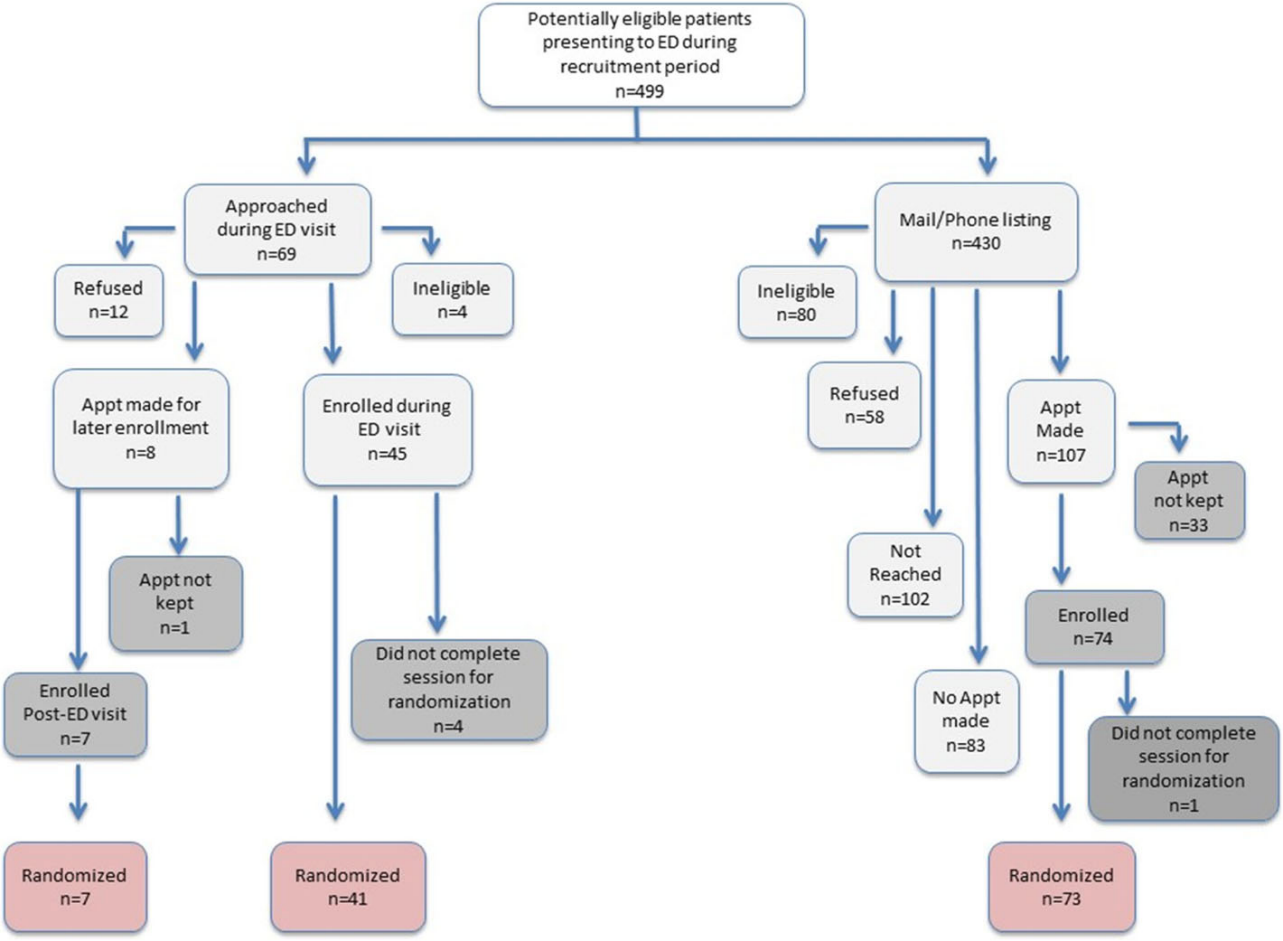
Recruitment and enrollment disposition for targeted population

Patients were recruited from October 2012 to October 2013. A recruitment flowchart is presented as Fig. 1. A total of 126 patients completed a baseline, of which 121 (96%) were randomized (targeted enrollment = 120). Of these, the majority of participants were African-American (86%), with the mean age of 15.4 years (±1.7 years). Although the randomization was not 1:1 due to the use of randomization strata, patient characteristics were balanced between treatment and control groups at baseline (Table 1).

**Table 1:** Pilot study baseline characteristics by randomization group

Characteristic/variable	All (*n* = 121)		Treatment (*n* = 65)		Control (*n* = 56)	
HFHS, % (*n*)	29.8	(36)	30.8	(20)	28.6	(16)
CHOM, % (*n*)	70.2	(85)	69.2	(45)	71.4	(40)
Age, mean (sd)	15.4	(1.7)	15.3	(1.6)	15.5	(1.8)
Male, % (*n*)	44.6	(54)	44.6	(29)	44.6	(25)
Hispanic/Latino, % (*n*)	8.3	(10)	7.7	(5)	8.9	(5)
African-American, % (*n*)	86.0	(104)	83.1	(54)	89.3	(50)
White, % (*n*)	3.3	(4)	4.6	(3)	1.8	(1)
American Indian, % (*n*)	1.6	(2)	3.1	(2)	0	(0)
Missing, % (*n*)^a^	0.8	(1)	1.5	(1)	0	(0)
Medicaid, % (*n*)	60.3	(73)	56.9	(37)	64.3	(36)
Home computer, % (*n*)	71.1	(86)	72.3	(47)	69.6	(39)
Provided SMS at baseline, % (*n*)	86.0	(104)	87.7	(57)	83.9	(47)
ACT score at baseline, median (range)	16.0	(6–25)	15.5	(7–24)	16.0	(6–25)
ASRDI at baseline, mean (sd)	2.8	(1.0)	2.9	(0.9)	2.6	(1.2)

*ACT* Asthma Control Test [15], *ASRDI* Asthma Self-Regulatory Development Interview [8]

^a^Missing race/ethnicity

### Compliance with study protocol and Internet access

Overall study compliance for completion of >3 sessions (64.5%) and 6-month follow-up (57%) was under the goal of 70%. At 12 months, completion was 89.3%. Consistently, the treatment group completed more events than controls (Table 2), but differences were not statistically significant. We conducted some exploratory, preliminary analyses regarding Internet access and compliance (data not shown in the table). For analysis purposes, compliance was defined as completion of >3 sessions + completion of the 6- or 12-month survey. As defined, 73.5% (89/121) of participants were compliant. Having Internet access at home was significantly associated with compliance, in that 80% (69/86) of youth with Internet access were compliant versus 57% (20/35) of those without access, OR = 3.04 (95% CI = 1.30–7.15), *p* = 0.0125 (data not shown in the table). Home Internet access was also associated with completion of the 6-month survey, in that 65% (56/86) of youth with Internet access completed the 6-month versus 37% (13/35) of those without access, OR = 3.13 (1.39–7.25), *p* = 0.006. This was not observed with the 12-month survey, in which 90.7% (78/86) of those with Internet access at home completed the 12-month survey versus 85.7% (30/35) of those without access, OR = 1.62 (0.45–5.41), *p* = 0.438.

**Table 2:** Pilot study compliance and 12-month primary and secondary outcomes

	Treatment (*n* = 65)		Control (*n* = 56)		Odds ratio	(95% CI)
Study compliance						
Completed >3 sessions, % (*n*)	66.2	(43)	62.5	(35)		
Completed 6-month survey, % (*n*)	60.0	(39)	53.6	(30)		
Completed 12-month survey, % (*n*)	92.3	(60)	85.7	(48)		
Primary outcome: utilization^a^						
>1 ED visit/hospitalization in follow-up year, % (*n*)	33.8	(22)	46.4	(26)	0.53	(0.24–1.15)
Secondary outcomes						
Change of ACT score from baseline, median (range)	2	(24)	2	(20)		
Functional status^b^ in the last 30 days						
>2 symptom days/week over 30 days						
Yes, % (*n*)	25.9	(15)	28.3	(13)	0.87	(0.36–2.11)
No, % (*n*)	74.1	(43)	71.7	(33)		
>2 school days missed/30 days (any reason)						
Yes, % (*n*)	9.6	(5)	5.3	(2)	1.85	(0.33–10.34)
No, % (*n*)	90.4	(47)	94.7	(36)		
>2 school or work days missed/30 days (asthma)						
Yes, % (*n*)	9.5	(4)	0	(0)	Not calculated	
No, % (*n*)	90.5	(38)	100	(29)		
ASRDI at 12 months, mean (sd)	3.1	(1.2)	2.7	(1.2)		
Targeted behaviors, % (*n*)						
Among patients reporting controller medication at session 1						
Adherent >5 days of the past 7 days, % (*n*)	62.1	(18)	50	(5)	1.62	(0.38–6.93)
Among patients reporting rescue inhaler at session 1						
Rescue inhaler available >5 days/7 days, % (*n*)	54.5	(24)	70.4	(19)	0.50	(0.18–1.36)

*ACT* Asthma Control Test [15], *ASRDI* Asthma Self-Regulatory Development Interview, self-report [8]

^a^Electronic health record abstraction

^b^Adjusted by baseline ACT score

### Outcomes and targeted behaviors

As shown in Table 2, the primary endpoint of ED visits was in the expected direction with a *p* = 0.15 and met a priori criteria for a potential intervention effect (i.e., *p* < 0.20). At 12-month follow-up, 33.8% of treatment teens had made an ED visit within the follow-up period, versus 46.4% of control teens, OR = 0.53 (0.24–1.15), *p* = 0.15. Results for secondary endpoints were more varied. Median changes in ACT scores were similar (median change = 2, *p* = 0.26); however, the range (maximum- minimum) was greater for the treatment group (24) versus the control group (20). Control teens reported fewer symptoms than treatment teens, but (ASRDI) scores were higher for the treatment group compared to the control group, 3.1 versus 2.7, respectively, and this difference was of borderline significance (*p* = 0.08).

At 12 months, among teens that had a controller medication at baseline, a higher percentage of treatment group teens self-reported adherence to a controller medication compared to control teens (62.1 versus 50%), OR = 1.62 (0.38–6.93), *p* = 0.71. In contrast, fewer treatment teens reported having a rescue inhaler nearby (54.5% in the treatment group versus 70.4% in the control group, *p* = 0.22).

Overall, 80.1% (97/121) of teens reported “never” smoking, 83.1% for the treatment group (54/65) and 76.8% (43/56) for the controls. Among those “ever” smoking at session 1, 8.3% (*n* = 10) smoked in the last 30 days: 10.8% (7/65) and 5.3% (3/50) for the treatment and control patients, respectively. At 12 months, this was reduced to 5.0% (3/60) and 2.1% (1/48) for the treatment and control, respectively.

In terms of patient safety, seven patients (three in the treatment group and four in the control group) had SAEs in a range of 23 to 118 days after study enrollment and required hospitalization. *n* = 2 had psychiatric disorders at days 27 and 118, while the remaining events could be categorized as a respiratory disorder (*n* = 2, at days 40 and 58), infection (*n* = 2 at days 23 and 177), and a gastrointestinal disorder (*n* = 1 at day 38). After review by the medical expert, none were found to be attributed to the study intervention.

## Discussion

We note several challenges to successful evaluation of this program using a RCT in terms of intervention delivery via the Internet, study compliance, and an early indication of effect. It is difficult to assess Internet access separately from study compliance. About 70% of youth in our study reported Internet access at home, and the association of this variable with study compliance was most evident with the 6-month survey. With additional assistance in finding computer resources, compliance with the 12-month survey was greatly improved. According to the latest PEW Report, 85% of African-American adolescents have smartphones and use them to go online daily. Given this level of online activity, technology-based health education remains a potential approach for urban, African-American teens. The version of Puff City used for this study may not have worked well on a smartphone. When this is the case, assistance with computer resources will be necessary for some youth.

We observed a modest potential intervention effect for the primary outcome of fewer ED visits, according to our a priori criteria for this pilot study of *p* < 0.20. Results for secondary outcomes varied. The ASRDI score and medication adherence supported findings for ED visits. The ASRI score assesses the patient’s ability to self-regulate asthma. Creators of the ASRI suggest that self-regulatory management of asthma may require “…changing fundamental beliefs about the illness, changing self-perceptions of vulnerability, and enhancing perceived efficacy for coping with symptoms…” [14]. Taken together, fewer ED visits, higher ASRDI scores, and better adherence could reflect a more prevention-oriented approach and better self-regulation of asthma, but small sample size and lack of consistent findings for secondary outcomes mean these results should be treated with caution. We must also consider that control programs were similar or superior to Puff City. Control programs were chosen by young adults working with our team, who were asked to select programs that were geared toward teens and that covered similar content.

We found it challenging to keep teens engaged in a program that has four sessions at least 1 week apart. Birthday cards and a quarterly newsletter were used to help us keep track of youth (for both the treatment and control groups) over the course of 1 year. Future analyses will determine the optimal number of sessions needed to obtain the desired effect.

A strength of this pilot study is the use of administrative databases and the EMR to collect information on the primary outcome of ED visits, as opposed to using self-report. Medical records were reviewed for all teens at both sites. HFHS and the Detroit Medical Center together see 77% of ED visits in Detroit [12] with patients frequently visiting these EDs interchangeably. Most EDs in the city of Detroit are affiliated with one of these health care systems. We have no evidence to support any likelihood that ED visits for teens randomized to the control group were systematically missed at a higher frequency.

In 2009, Boyd et al. published a Cochrane Review on interventions targeting youth that had attended the ED for asthma and with the goal of reducing the risk of subsequent visits. In 21 of the 38 studies, participants were recruited at the time of the emergency department/hospital admission for asthma. Overall, the interventions were successful in reducing ED visits, risk ratio = 0.73 (95% CI = 0.65–0.81), although the authors were unable to discern if initiation of the intervention in the ED was superior to initiation within 12 months of the index ED visit [16]. Seventeen of the 38 studies reviewed recruited youth from low-income and disadvantaged areas, but only one study recruited adolescents exclusively, and this study suffered from high attrition (52%) [16, 17].

Factors such as exposure to community violence, caregiver mental health, caregiver social support, and life stress, as well as lack of insurance and access to medical care, have all been associated with use of the ED for asthma [18–21]. As has been mentioned by several experts, addressing social issues could be as important in controlling asthma as learning to use an inhaler in the correct way [18–20]. Lack of Internet access at home could be an indicator of other economic and social disadvantages that make study participation difficult. Boyd et al., in the Cochrane Review of emergency department interventions for asthma, suggested that variable treatment effects should be anticipated if access to primary care and other social factors (e.g., community violence) varied between study samples [16].

## Conclusions

In an earlier publication, we share recruitment challenges that must be overcome when conducting a trial of Puff City in the ED [11]. The present analysis suggests that study compliance and the factors associated with compliance could be major challenges as well. Our results suggest this barrier can be overcome with additional support in helping youth find Internet resources. A mobile version of Puff City that works on a smartphone may improve compliance for the 30% of teens with Internet access at home. Future analyses could also determine the optimal number of sessions needed to see a significant intervention effect. Intervening in the ED at the time of an acute asthma exacerbation could be an effective strategy in reducing subsequent ED visits and promoting better asthma control. This pilot work lays the foundation for conducting a large pragmatic trial with an age-appropriate and culturally optimized technology-based intervention to reduce the burden of asthma in adolescents.

## Abbreviations

ASRDI: Asthma Self-Regulatory Development Interview
CHOM: Children’s Hospital of Michigan
CI: Confidence interval
ED: Emergency department
EMSTAT/FIRSTNET: Remote electronic surveillance and triage systems
HFHS: Henry Ford Health System
IRB: Institutional Review Board
OR: Odds ratio
PaO2: Arterial oxygen pressure
PCO2: Partial pressure of carbon dioxide
PEF: Peak flow
RCT: Randomized controlled trial
SaO2: Arterial oxygen saturation
SES: Socioeconomic status
UM-CHCR: University of Michigan Center for Health Communications Research
